# *Chlorella vulgaris* Modulates Gut Microbiota and Induces Regulatory T Cells to Alleviate Colitis in Mice

**DOI:** 10.3390/nu15153293

**Published:** 2023-07-25

**Authors:** Priyanka Velankanni, Seok-Ho Go, Jong Beom Jin, Jin-Soo Park, Sunhee Park, Su-Bin Lee, Ho-Keun Kwon, Cheol-Ho Pan, Kwang Hyun Cha, Choong-Gu Lee

**Affiliations:** 1 Natural Product Informatics Research Center, Korea Institute of Science and Technology (KIST), Gangneung 25451, Republic of Korea; priyanka@kist.re.kr (P.V.); jojin@okstate.edu (J.B.J.); jinsoopark@kist.re.kr (J.-S.P.); psh@kist.re.kr (S.P.); 2 Department of Marine Food Science and Technology, Gangneung-Wonju National University, Gangneung 25457, Republic of Korea; 3 Department of Preventive Medicine, School of Medicine, Kangwon National University, Chuncheon 24341, Republic of Korea; logos@skai.or.kr; 4 Department of Microbiology and Immunology, Yonsei University College of Medicine, Seoul 03722, Republic of Korea; leesb929@yuhs.ac (S.-B.L.); HK@yuhs.ac (H.-K.K.); 5 Division of Bio-Medical Science and Technology, Korea Institute of Science and Technology (KIST), University of Science and Technology, Seoul 02792, Republic of Korea; 6 Department of Convergence Medicine, Wonju College of Medicine, Yonsei University, Wonju 26493, Republic of Korea

**Keywords:** *Chlorella vulgaris*, regulatory T cells, gut microbiota, short-chain fatty acids, inflammatory bowel disease

## Abstract

*Chlorella vulgaris* (*C. vulgaris*) is unicellular green algae consumed worldwide as a functional food. The immune stimulatory function of *C. vulgaris* is known; however, no study has elucidated its immune regulatory potential and associated microbiome modulation. In the current study, we aimed to validate the immune regulatory role of *C. vulgaris* mediated through two mechanisms. Initially, we assessed its ability to promote the expansion of the regulatory T cell (Treg) population. Subsequently, we investigated its impact on gut microbiota composition and associated metabolites. The supplementation of *C. vulgaris* altered the gut microbiota composition, accompanied by increased short-chain fatty acid (SCFAs) production in mice at homeostasis. We later used *C. vulgaris* in the treatment of a DSS-induced colitis model. *C. vulgaris* intervention alleviated the pathological symptom of colitis in mice, with a corresponding increase in Treg levels. As *C. vulgaris* is a safe and widely used food supplement, it can be a feasible strategy to instigate cross-talk between the host immune system and the intestinal flora for the effective management of inflammatory bowel disease (IBD).

## 1. Introduction

The incidence of inflammatory bowel disease (IBD) has stabilized in the West; however, its prevalence remains high [1]. Studies from newly industrialized countries have shown an enhanced IBD incidence that is associated with the increased westernization of dietary habits [2]. IBD is a multifaceted disease characterized by an impaired barrier, increased intestinal permeability, and dysregulated mucosal immune response. These factors disrupt the fine line between tolerance and inflammation [3]. Conventionally, there are two main categories of therapeutics for IBD treatment: (1) anti-inflammatory or immunosuppressive agents and (2) biological agents. Examples of classic IBD therapies include 5-aminosalicylates, azathioprine, methotrexate, cyclosporine-A, infliximab, adalimumab, and golimumab [4]. However, these drugs are only modestly successful; hence, safer and more natural alternatives for the long-term treatment of IBD are sought.

The pathogenesis of IBD is complicated and involves several variables, including diet, immunologic factors, environmental factors, infectious agents, genetic susceptibility, and the microbiome, which mediate dysregulated immune responses against the microbiome and allergens [4]. The adaptive immune system is a primary contributor to mucosal damage in IBD, which is mediated either by aberrant pro-inflammatory cytokines produced by T helper (Th) subsets or by a dysfunctional regulatory compartment [5]. Treg cells are vital players in the immune regulatory subset and are typically identified by the presence of markers such as CD4, CD25, and the key transcription factor FOXP3 [6]. Defects in Tregs and their mediators are central to IBD pathogenesis; hence, they are the most critical targets of drugs for IBD treatment [7]. The diet-based regulation of Tregs is a probable strategy for managing IBD. For example, administering an Aronia-supplemented diet to colitis mice effectively restored the Treg/Th17 balance and alleviated colitis symptoms [8].

Additionally, the gut microbiota is considered a central determinant in the pathogenesis of IBD. Gut dysbiosis and aberrant microbial sensing are commonly observed in IBD patients [9]. Metabolites produced by the gut microbiota can indicate dietary changes, microbial composition, and host physiological processes [10]. These microbial metabolites are crucial in facilitating host–microbe communication [11]. Microbial metabolites have a significant impact on intestinal health regulation [12]. Metabolites present in the gut are often considered markers of dietary factors and microbial interplay in the gut [13]. Patients with IBD exhibit a decrease in total short-chain fatty acids (SCFAs) levels [14].

*Chlorella vulgaris* (*C. vulgaris* or CV) is rich in vital macro- and micronutrients and is considered a safe and well-known functional food. It is a widely commercialized nutritional supplement consumed worldwide, particularly in Asia [15]. Multiple studies have suggested the immunomodulatory role of *C. vulgaris*; hence, the consumption of *C. vulgaris* can be a potential dietary therapy for treating various autoimmune diseases [16]. In a murine asthma model, supplementation with *C. vulgaris* inhibited the Th2 response, substantiated by a drop in the levels of Th2 associated cytokines such as IL-4, IL-5, and IL-13 [17]. *C. vulgaris* reduced the mRNA expression levels of Th2 and Th1 cytokines, namely IL-4 and IFN-γ, in an atopic dermatitis-induced mouse [18]. In contrast, some reports suggest the role of *C. vulgaris* in the enhancement of Th1- and Th2-mediated responses [15,19]. In a clinical study, supplementation with *C. vulgaris* resulted in elevated serum levels of IL-12, IL-1β, and IFN-γ and enhanced the natural killer cell activity, suggesting an immunostimulatory effect of *C. vulgaris* [20].

Dietary habits determine the diversity and composition of the gut microbiota [21]. The gut microbiome decomposes dietary fibers and indigestible oligosaccharides in food and synthesizes SCFAs, such as butyric acid, propionic acid, and acetate [22]. SCFAs are often associated with the expansion of colonic Tregs [23]. An earlier study showed that dietary supplementation with *C. vulgaris* impeded the development of intestinal inflammation in Atlantic salmon [24]. However, no studies have clarified the effect of *C. vulgaris* in a colitis model and its immune regulatory role mediated by microbiome modulation. The administration of *C. vulgaris* can be a potential dietary therapeutic approach against the management of IBD, as it is rich in various micro- and macronutrients essential for expanding a regulatory environment in the colon mediated by a healthy microbiota composition. Hence, this study investigated the prophylactic effect of *C. vulgaris* in a DSS-induced colitis model and inspected its immunomodulatory effects and effects on microbiome composition and metabolites.

## 2. Materials and Methods

### 2.1. Animals

C57BL/6 female mice (6 weeks old) were housed in a specific pathogen-free facility with alternate 12 h light and dark cycles in a controlled environment with ambient temperature (23 ± 2 °C) and 40% humidity. The animals were caged in groups of 6 or fewer and had unrestricted access to pellet-based food and water. The Korea Institute of Science and Technology’s International Animal Care and Use Committee approved animal experiments (Approval code: KIST-IACUC-2022-080).

### 2.2. Chlorella vulgaris Treatment in Homeostatic and Experimental Colitis Mice

Dried and powdered *Chlorella vulgaris* hot water extract was procured from Daesang Corporation (Seoul, Korea). For the homeostatic condition study, C57BL/6 mice were divided into two groups: the control (Cont; n = 11) and *C. vulgaris* treated (CV; n = 13). The CV group received oral gavage of *C. vulgaris* for a duration of 3 weeks. To investigate the effect of *C. vulgaris* in the DSS-induced colitis mice, C57BL/6 mice were randomly divided into four groups: control group (Cont or mock; n = 5), *C. vulgaris* treated (CV; n = 4), DSS-induced colitis without *C. vulgaris* treatment (DSS; n = 6) and DSS-induced colitis with *C. vulgaris* treatment (DSS + CV; n = 9). The mice in the CV and the DSS + CV group were orally administered 2 g/kg *C. vulgaris* extract for 3 weeks. Acute colitis was induced in the DSS and DSS + CV group by means of administering 2.5% (*w*/*v*) colitis grade DSS (160110, MP Biomedicals, OH, USA) in autoclaved drinking water for 7 days. Whereas the animals in the Cont and CV group received drinking water without DSS. The severity of colitis was then assessed through various macroscopic and microscopic evaluation methods.

### 2.3. Evaluation of Disease Activity Index (DAI)

Experimental animals were monitored daily for changes in body weight, fecal consistency, and rectal bleeding after DSS treatment. DAI was evaluated based on the mean of the three following parameters, (a) body weight change (<1% loss: 0 point; 1–5% loss: 1 point; 5–10% loss: 2 points, 10–20% loss: 3 points; >20% loss: 4 points), (b) stool consistency, and (c) rectal bleeding (normal: 0 point; soft stool+ positive hemoccult: 1 point; very soft stool with traces of blood: 2 points; watery stool with visible rectal bleeding: 3 points; diarrhea with gross bleeding: 4 points). The humane end-point was established at DAI = 3.

### 2.4. Histology and Scoring

The fecal contents of the colon tissues were flushed using ice-cold PBS. Subsequently, the colon tissue was fixed in 4% (*w*/*v*) formalin, paraffin-embedded, and sectioned. The sections were subjected to hematoxylin and eosin (H&E) staining and graded by an experienced pathologist in a blinded manner. The histological index was determined based on the parameters described previously [25].

### 2.5. Cell Isolation and Flow Cytometry

Mononuclear cells were isolated from mesenteric lymph nodes (mLNs) and spleen. The cells isolated from the spleen samples were then subjected to RBC lysis using RBC lysis buffer (Biolegend, San Diego, CA, USA) to obtain the lymphocytes. The cells were labeled with the Fixable Viability Dye eF780 (eBioscience, San Diego, CA, USA) to identify and exclude the non-viable cells from further analysis selectively. The cells were then subjected to FITC-labeled anti-mouse CD4 antibody (Biolegend, San Diego, CA, USA), PE-Cy7 labeled anti-mouse CD8a (Tonbo Bioscience, San Diego, CA, USA), and APC labeled Nrp1 (R&D SYSTEMS, Minneapolis, MN, USA) for surface staining. After fixation and permeabilization, the cells were stained with PE-labeled Foxp3 Monoclonal antibody (FJK-16s) (eBioscience, San Diego, CA, USA) and APC-labeled Rorgt (eBioscience, San Diego, CA, USA). The stained cells were then analyzed using a BD FACSVerse^TM^ flow cytometer (BD Bioscience, San Jose, CA, USA). The data analysis was conducted using Flow Jo 10.0 (Three Star, Ashland, OR, USA).

### 2.6. Microbial Community Analysis

The total genomic DNA was extracted from the cecum using the QIAamp^®^ Fast DNA Stool Mini Kit (Qiagen, Hilden, Germany), and an additional bead-beating step was included. The isolated genomic DNA was amplified for the V3–V4 hypervariable region of the 16S rRNA gene by the universal primers 341F and 805R. The resulting PCR products were purified and sequenced as previously described [26]. The raw reads were processed using the QIIME 2-DADA2 pipeline (v2023.2), which involved demultiplexing, quality filtering, and assembly [27,28]. To assign classifications to filtered reads at the bacterial taxonomic levels, Naïve Bayes classifier was trained using the V3-V4 16S rRNA region, the 341/805R primer set, and the SILVA v138 (99%) database. Microbial composition and diversity were analyzed using MicrobiomeAnalyst 2.0 [29]. Non-metric multidimensional scaling (NMDS) plots were generated according to the Bray–Curtis distance matrix to visualize the variability in the microbiota composition among groups. To identify the differentially abundant taxa at the genus level between the groups, linear discriminant analysis effect size (LEfSe) was applied with a significance level α = 0.05 and a minimum linear discriminant analysis (LDA) score of 2 [30].

### 2.7. Cecal SCFA Analysis

The cecal samples were weighed and processed with oxalic acid (0.1 mol/L) and sodium azide (40 umol/L), and were analyzed with a 300-MS Bruker Gas chromatographic system with a Flame ionization detector (FID) and later quantified by comparing the peak areas to the standards. During the experiment, the oven temperature was kept constant at 170 °C while the injector and detector temperatures were adjusted to 225 °C. A Nukol column (Supelco), a fused silica capillary column measuring 30 m × 0.25 mm with a film thickness of 0.25 µM, was used for the analysis.

### 2.8. Statistical Analysis

All statistical analysis was conducted using GraphPad Prism (version 9.0; GraphPad Software, La Jolla, CA, USA). Differences were deemed significant at *p* 0.05 after comparing each group using Student’s *t*-test, Mann–Whitney test, and one- or two-way ANOVA corrected for multiple comparisons with the Sidak test.

## 3. Results

### 3.1. C. vulgaris Promotes Treg Generation In Vivo

**Figure 1:**
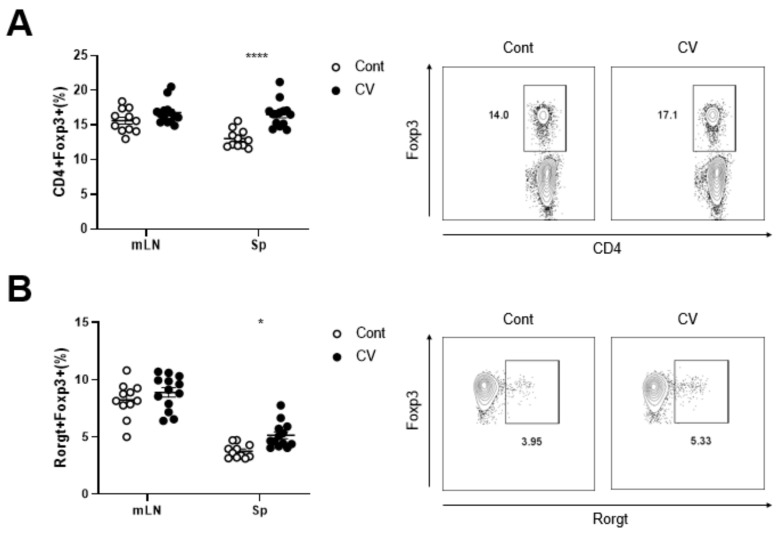
CV supplementation upregulated the Treg population in lymphoid tissues. (**A**) Percentage of Tregs in mLN and spleen and a representative frequency plot of Tregs. (**B**) Percentage of Rorγt^+^ in CD4^+^Foxp3^+^ Tregs in mLN and spleen and a representative frequency plot. Statistical significance was determined using 2-way ANOVA (*, *p* < 0.05; ****, *p* < 0.0001).

CV was orally fed to mice for three weeks, and the lymphoid tissues were analyzed for change in the Treg population. As anticipated, the CV treatment significantly increased the CD4^+^Foxp3^+^ Treg percentage in the spleen (Figure 1A). In addition, we also observed an increase in the levels of Rorγt^+^Foxp3^+^ regulatory cells in the spleen of the CV-treated mice (Figure 1B). Rorγt^+^Foxp3^+^ T cells can be regarded as a more stable regulatory effector lineage and can elicit an enhanced suppressive effect during gut-specific immune response [31,32]. These findings suggest that CV treatment can result in the quantitative enhancement of the Treg population in the lymphoid tissue; in particular, the increase in the levels of Treg in the spleen and mLNs suggest that the CV could regulate and dampen the excessive immune response to maintain immune homeostasis.

### 3.2. C. vulgaris Treatment Altered Cecal Bacterial Diversity and Composition

**Figure 2:**
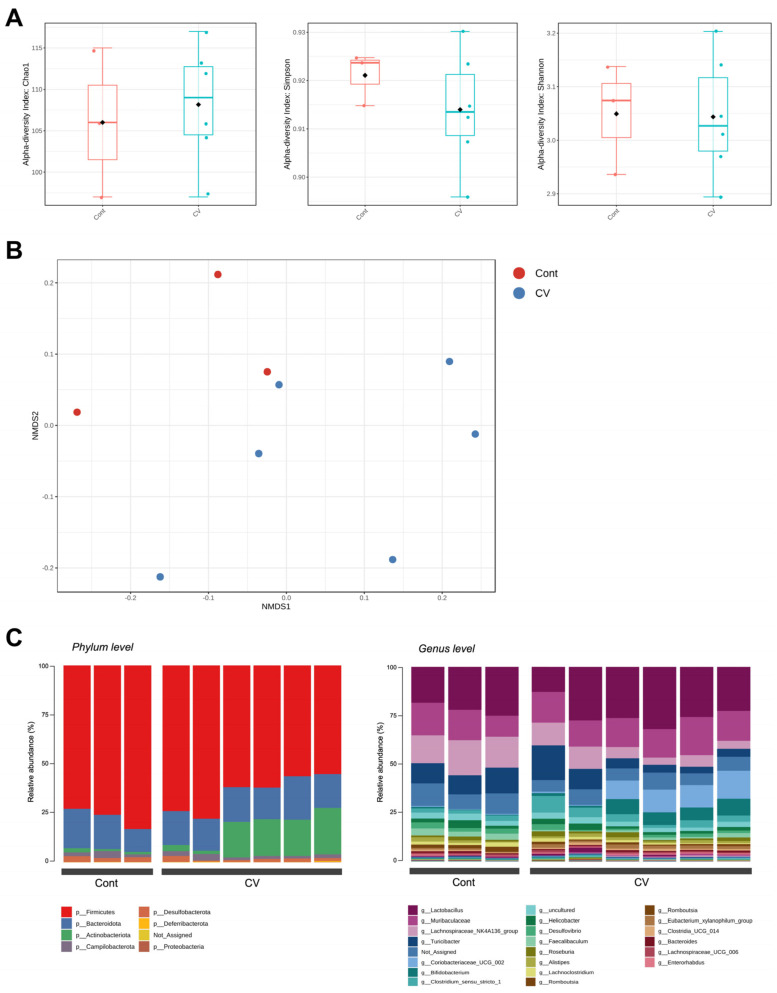
Administration of CV-modulated gut microbiota composition. (**A**) Alpha diversity boxplots (Chao1, Simpson, and Shannon indices). The dots in the plots represent the data points and diamonds represent the mean. (**B**) Non-metric multidimensional scaling (NMDS) plot of the gut microbiota. (**C**) Phylum- and genus-level microbiota composition in the cecum.

Gene sequencing analysis of the 16S rRNA gene for the V3-V4 variable region of cecal bacteria isolated from control and CV-fed mice at homeostatic conditions was conducted to determine whether CV treatment could alter the microbiome composition. CV administration did not alter the alpha diversity statistically, as measured by the Chao1, Simpson, and Shannon indices (Figure 2A). However, CV treatment affected beta diversity; the NMDS plots show that the microbial clusters are clearly separated between the two groups. The farther the points, the more microbial community composition differences (Figure 2B). We further analyzed the gut microbial composition both at the phylum and genus levels. Feeding the CV group resulted in an increased abundance of Actinobacteria phylum, which seemed to be due to the notable increase in *Coriobacteriaceae UCG 002* and *Bifidobacterium* at the genus level (Figure 2C).

### 3.3. C. vulgaris Treatment Augments SCFA Levels

**Figure 3:**
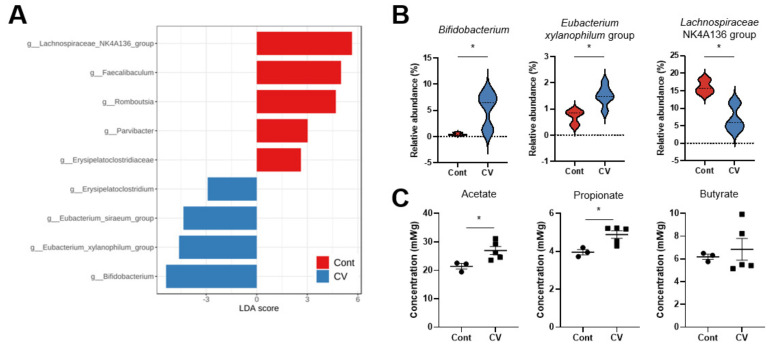
Administration of CV augments the abundance of SCFA-producing bacteria. (**A**) Histogram of LDA scores computed for differentially abundant bacteria between the CV-treated and non-treated controls. (**B**) Violin plot showing the mean and distribution pattern of the relative abundance of the *Bifidobacterium* and *Eubacterium xylanophilum* group, and the *Lachnospiraceae NK4A36* group between the control and the CV-treated group. (**C**) CG-FID quantification of acetate, propionate, and butyrate in the cecal contents. Statistical significance was determined using the Mann-Whitney test and Student’s *t*-test (*, *p* < 0.05).

Many exogenous and endogenous factors affect the gut microbiome and microbial metabolites, such as SCFAs. Numerous gut-inhabiting bacteria are involved in synthesizing SCFAs by utilizing dietary fibers as the substrates [33]. In our study, the relative abundance of the *Bifidobacterium* and *Eubacterium xylanophilum* group increased due to the CV treatment (Figure 3A,B). To further clarify the effect of CV on levels of bacterial metabolites, we used GC-FID to analyze the levels of cecal SCFAs in mice after three weeks of CV intervention. Acetate and propionate levels increased significantly due to CV intake, and although we could not discern a significant increase in butyrate levels, an upward trend was observed (Figure 3C). Lower levels of butyrate can be due to the reduction in the *Lachnospiraceae NK4A136* group, a well-known butyrate producer [34] (Figure 3B). All these results imply that the administration of CV led to a comprehensive alteration in both gut microbiota composition and SCFA levels.

### 3.4. C. vulgaris Ameliorates DSS Induced Colitis

**Figure 4:**
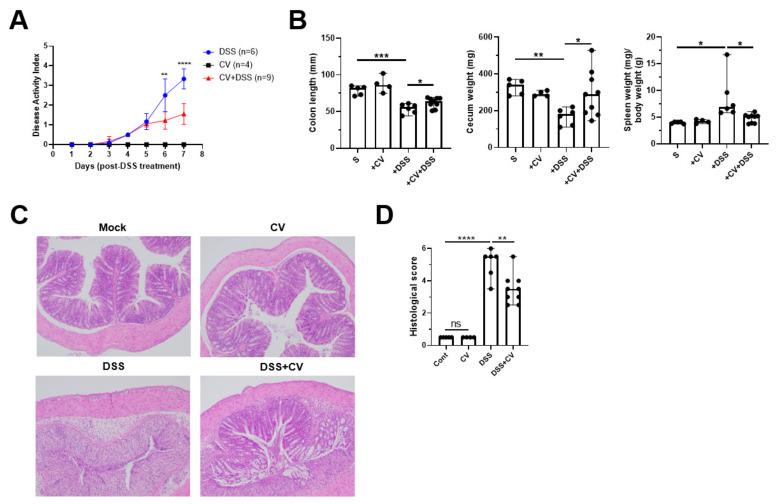
Supplementation of CV attenuated DSS-induced colitis. (**A**) The disease activity index post DSS treatment (**B**) Colon length, cecum weight, spleen weight/body weight ratio. (**C**) Representative H&E-stained colon sections (magnification: 100X). (**D**) The histological assessment of colitis severity. Statistical significance was determined using Student’s *t*-test (*, *p* < 0.05; **, *p* < 0.01; *** *p* < 0.001; ****, *p* < 0.0001, ns—non-significance).

Due to the effect of CV in the induction of Tregs and the ability to modify the gut microbial and metabolite composition, we sought to investigate its anti-colitic effect in DSS colitis mice. As expected, the DSS group displayed a dramatic increase in DAI scores; however, the CV-treated mice displayed a significant reduction in DAI scores (Figure 4A). In addition, CV treatment restored the shortening of colon length, an essential characteristic of severe colonic inflammation to a certain extent when compared with the non-treated DSS group (Figure 4B, left). Treatment with CV resulted in the restoration of cecal weight in colitis mice (Figure 4B, middle). Furthermore, we observed a significant reduction in the spleen swelling as characterized by a reduction in the spleen weight/body weight ratio due to the CV treatment in the colitis mice (Figure 4B, right). Finally, the histology of the colon revealed that the administration of CV reduced the histological score by improving the mucosal damage, destruction of crypts and goblet cells, and reducing the infiltration of inflammatory cells (Figure 4C,D). Our results thus highlight how CV administration effectively ameliorated various signatures of the colitis mice.

### 3.5. C. vulgaris Treatment Restores Tregs Levels in Colitis Mice

**Figure 5:**
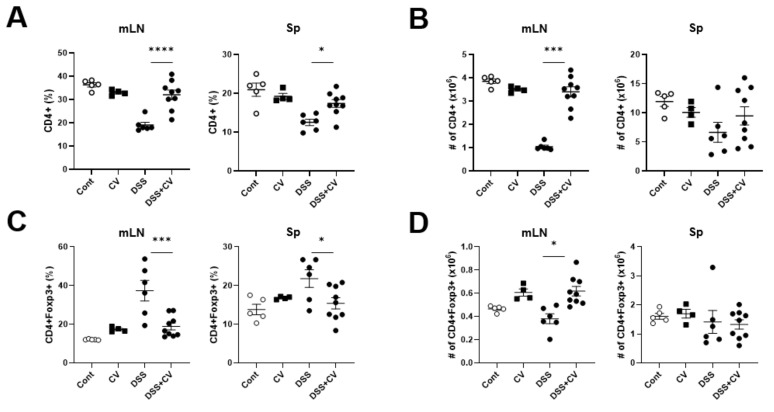
CV increases the Treg population in colitis mice. (**A**) The percentage of CD4+ T cells in mLN and spleen. (**B**) The absolute cell numbers of CD4+ T cells in mLN and spleen. (**C**) The percentage of CD4+ Foxp3+ cells in mLN and spleen. (**D**) The absolute cell numbers of CD4+ Foxp3+ cells in mLN and spleen. Statistical significance was determined using ordinary 1-way ANOVA (*, *p* < 0.05; *** *p* < 0.001; ****, *p* < 0.0001).

To validate the immune regulatory potential of CV in colitis mice, we evaluated the percentage of Tregs in the spleen and mLN. The imbalance in the levels of Tregs is pivotal in the development of IBD, and the role of the microbiota in the maintenance of Tregs is indispensable. Dietary components dictate the gut microbiota’s vital composition [35,36]. Colitis induction resulted in an overall moderation of the percentage and the absolute numbers of the CD4^+^ population in mLN and the spleen (Figure 5A,B). Contrarily, CV administration resulted in an overall increment in the absolute number of the CD4^+^Foxp3^+^ T cells in the mLNs (Figure 5D). However, the decrease in the proportion of CD4^+^Foxp3^+^ T cells (Figure 5C) can be attributed to the fact that DSS treatment led to a moderation in the total percentage of CD4+ T cells in the spleen and the mLN (Figure 5A). These findings imply that oral CV administration protected against IBD by promoting immune tolerance via immune regulatory mechanisms mediated by Tregs.

## 4. Discussion

CD4+ T cell dysfunction is a common mechanism responsible for initiating IBD. Aberrant immune responses are generally associated with defective immune regulatory mechanisms responsible for resolving and controlling the functions of other effector T cells [37]. Foxp3+ Tregs in IBD patients are more likely to co-express the cytokines and transcription factors of the effector subtypes [38,39]; hence, the restoration of the stable Foxp3-expressing Treg population can be successful in alleviating IBD. Dietary agents such as EGCG, a green tea polyphenol, can induce Foxp3 expression [40]. Algae is abundant in a variety of health-enhancing elements such as amino acids, vitamins, antioxidants, functional carbohydrates and other valuable bio-active compounds that contribute to the overall well-being [41]. Evaluating dietary agents that can effectively modulate immune function is critical for implementing dietary interventions for managing immune disorders. Regarding the biological safety and toxicity of CV, it is reported that no acute toxicity was detected in mice treated with 2000 mg/kg of CV [42]. Our current study demonstrated that during homeostasis, CV administration induced the systemic expansion of Treg cells with a concomitant increase in the MFI of Foxp3, which is often associated with the higher suppressive activity of Tregs. CV administration supposedly promotes Foxp3 stability and consequently improves the functionality of Tregs. In addition, an increased percentage of Foxp3 was observed in colonic lamina propria (data not shown), which could be due to the microbial fermentation of indigestible dietary fibers in the lower gastrointestinal tract, such as the colon. Therefore, the oral administration of CV directly and systematically promotes an immune regulatory environment.

Another important consequence of dietary intervention with CV is increased gut microbiota diversity. Analysis of the relative abundance of the bacterial taxonomic groups at the end of the three weeks of CV supplementation revealed an increase in cecal commensal diversity. As defined by the Chao1, Simpson, and Shannon indices, alpha diversity did not show a statistical increase in diversity among the CV- and non-treated groups. However, the principal coordinate analysis plots generated from the calculated beta diversity displayed a shift in bacterial clustering between the control and CV-treated groups. A genus-level increase in *Coriobacteriaceae UCG 002* and *Bifidobacterium* levels was observed. *Bifidobacterium* can also shape the Treg population by promoting the generation of functional Treg cells [43,44,45]. One of the key physiological functions of the gut microbiota is to metabolize dietary factors into functional metabolites such as SCFAs. SCFAs act as an effective signaling molecule for regulating the function and number of Tregs; furthermore, SCFAs regulate the recruitment and extrathymic conditioning of Tregs in the gut, making SCFAs a critical molecule that can mediate the communication between the gut microbiota and the host immune system [22,23]. *Coriobacteriaceae UCG 002* is linked with the increase in SCFA concentration [46]. The microbes belonging to the *Bifidobacterium* are among the most commonly used probiotics owing to their wide range of beneficial effects, and they are reported to promote SCFA production [47,48]. In our study, increased levels of *Eubacterium xylanophilum* were observed, a well-known butyrate-producing bacterium [49]. Taken together, it is evident that CV administration resulted in microbial modulation, accompanied by increased SCFA production, eventually resulting in Treg expansion.

Owing to Treg induction and the microbial modulating effect of CV, we used CV to evaluate its prophylactic effect in a DSS colitis model. Gut microbial dysbiosis and dysregulation of Tregs are commonly observed in IBD patients. As seen from the results, CV effectively ameliorated experimental colitis, as demonstrated by the reduction in the DAI and spleen index, the shortening of colon length, and the histological score. These results suggest that CV alleviated tissue deterioration and inflammation are associated with colitis. We further analyzed the Treg-inducing effect of CV. As anticipated, an increase in the Treg cell percentage was observed, especially in mLNs, the gut-draining lymph node, which indicates the direct effect of CV in promoting immune tolerance towards enteric antigens in colitis mice.

However, we needed to clarify the exact mechanism by which microbiome modulation and immune responses are interlinked. However, we speculate that Treg expansion is mediated by microbiome modulation, as we could see increased Tregs in the lamina propria of the colon after CV administration, and it is known that the colon is the primary site for SCFA synthesis mediated by the microbial fermentation of the undigestible dietary fibers. The possible mechanism by which CV potentiates Treg expansion could be mediated through the major SCFA receptors, GPR41 and GPR43, that mediate various signaling pathways and gene regulation in various immune cells [50,51]. However, SCFAs tend to regulate immune cells via various other mechanisms beyond the cell surface GPRs, such as passive diffusion across the immune cell membrane, via sodium-coupled transporters or GPCR-independent HDAC inhibition, mainly by butyrate and propionate, resulting in Treg expansion [52,53,54]. Therefore, additional experimental investigations are required to unravel the mechanism of action by which CV administration induces Treg expansion.

Additional efforts were made to identify the molecule responsible for the Treg-inducing effect of the CV extract. However, the molecule isolated from the CV extract did not exhibit Treg-inducing effects (data not shown). Taken together, we can conclude that the Treg-inducing effect of the CV extract is not due to a single compound, but instead, its effects are mediated by the enormous amounts of proteins, dietary fibers, and all other macro- and micronutrients present in the extract.

## 5. Conclusions

Our research demonstrates that *C. vulgaris* plays a significant role as an immune regulatory agent. This is supported by our findings showing an increase in the population of CD4+ regulatory T cells and an elevated population of Rorγt + Foxp3+ cells in the homeostatic mice model. Additionally, the administration of *C. vulgaris* resulted in changes in the composition of gut microbiota, leading to enhanced synthesis of short-chain fatty acids. Moreover, *C. vulgaris* exhibited protective effects against DSS-induced colitis by modulating the immune profiles, particularly by enhancing the levels of regulatory T cells. Investigating specific components of *C. vulgaris* extract that contribute to T cell homeostasis could provide valuable insights for potential therapy for inflammatory bowel disease.

## Data Availability

All data are available upon request to the corresponding author.
